# Supplementary material for: Measuring the impact of social-distancing, testing, and undetected asymptomatic cases on the diffusion of COVID-19
Source: PLoS One. 2022 Aug 25;17(8):e0273469. doi: 10.1371/journal.pone.0273469 (PMC9409553; doi:10.1371/journal.pone.0273469)
Supplement: Attachment — Submitted filename: Response to Reviewers.docx [file pone.0273469.s002.docx]

**REBUTTAL LETTER**

July 18, 2022

PLOS ONE revision PONE-D-21-22695

## Dr. Siew Ann Cheong

Academic Editor

PLOS ONE

Dear Dr. Cheong:

I thank Dr. Cheong and the two reviewers for their comments on my manuscript.

Please check below my answer to each point raised by the editor and reviewers. I wish that I agreeably replied all of them, and that the revised manuscript will be suitable to be published in PLOS ONE.

Sincerely,

Seungyoo Jeon

**Journal Requirements**

When submitting your revision, we need you to address these additional requirements.

1. Please ensure that your manuscript meets PLOS ONE's style requirements, including those for file naming. The PLOS ONE style templates can be found at 
https://journals.plos.org/plosone/s/file?id=wjVg/PLOSOne_formatting_sample_main_body.pdf and 
https://journals.plos.org/plosone/s/file?id=ba62/PLOSOne_formatting_sample_title_authors_affiliations.pdf

***Answer) I checked the templates and modified the manuscript to meet the PLOS ONE style requirements.***

2. We suggest you thoroughly copyedit your manuscript for language usage, spelling, and grammar. If you do not know anyone who can help you do this, you may wish to consider employing a professional scientific editing service.

Whilst you may use any professional scientific editing service of your choice, PLOS has partnered with both American Journal Experts (AJE) and Editage to provide discounted services to PLOS authors. Both organizations have experience helping authors meet PLOS guidelines and can provide language editing, translation, manuscript formatting, and figure formatting to ensure your manuscript meets our submission guidelines. To take advantage of our partnership with AJE, visit the AJE website (http://learn.aje.com/plos/) for a 15% discount off AJE services. To take advantage of our partnership with Editage, visit the Editage website (www.editage.com) and enter referral code PLOSEDIT for a 15% discount off Editage services.  If the PLOS editorial team finds any language issues in text that either AJE or Editage has edited, the service provider will re-edit the text for free.

Upon resubmission, please provide the following:
 
The name of the colleague or the details of the professional service that edited your manuscript

A copy of your manuscript showing your changes by either highlighting them or using track changes (uploaded as a *supporting information* file)

A clean copy of the edited manuscript (uploaded as the new *manuscript* file)

***Answer) According to the suggestion, I employed a professional scientific editing service, Editage. The job code was “JDQCN_1”, and the revised manuscript was edited by “Bruce_8”. I uploaded two types of manuscripts – a clean copy of the edited manuscript and a copy of the manuscript showing my changes by using track changes.***

3. In your Data Availability statement, you have not specified where the minimal data set underlying the results described in your manuscript can be found. PLOS defines a study's minimal data set as the underlying data used to reach the conclusions drawn in the manuscript and any additional data required to replicate the reported study findings in their entirety. All PLOS journals require that the minimal data set be made fully available. For more information about our data policy, please see http://journals.plos.org/plosone/s/data-availability.

Upon re-submitting your revised manuscript, please upload your study’s minimal underlying data set as either Supporting Information files or to a stable, public repository and include the relevant URLs, DOIs, or accession numbers within your revised cover letter. For a list of acceptable repositories, please see http://journals.plos.org/plosone/s/data-availability#loc-recommended-repositories. Any potentially identifying patient information must be fully anonymized.

Important: If there are ethical or legal restrictions to sharing your data publicly, please explain these restrictions in detail. Please see our guidelines for more information on what we consider unacceptable restrictions to publicly sharing data: http://journals.plos.org/plosone/s/data-availability#loc-unacceptable-data-access-restrictions. Note that it is not acceptable for the authors to be the sole named individuals responsible for ensuring data access.

We will update your Data Availability statement to reflect the information you provide in your cover letter.

***Answer) I uploaded my study’s minimal underlying data set. Since the data set are fully open access under the Creative Commons BY license, there are no ethical or legal restrictions to sharing the data publicly.***

4. Please upload a new copy of Figure 5 as the detail is not clear. Please follow the link for more information: https://blogs.plos.org/plos/2019/06/looking-good-tips-for-creating-your-plos-figures-graphics/" https://blogs.plos.org/plos/2019/06/looking-good-tips-for-creating-your-plos-figures-graphics/

***Answer) I uploaded a new copy of Figures 6-8 (Figure 5 in the old version) by referring to the linked pages.***

5. Please ensure that you refer to Figures 1-5 in your text as, if accepted, production will need this reference to link the reader to the figure.

***Answer) I referred to Figures 1-8 (Figures 1-5 in the old version) in the revised manuscript.***

6. We note you have included a table to which you do not refer in the text of your manuscript. Please ensure that you refer to Tables 1-3 in your text; if accepted, production will need this reference to link the reader to the Table.

***Answer) I referred to Tables 1-7 (Tables 1-3 in the old version) in the revised manuscript.***

7. Please upload a copy of Supporting Information Figures S1-S5 and S1-S3 Table which you refer to in your text on page 23.

***Answer) Since Figures S1-S5 and Tables S1-S3 should be included to the manuscript, I added Figures 1-8 and Tables 1-7 to the accurate positions in the manuscript rather than uploading a copy of Supporting Information. Figures were separated and converted to TIFF via the Preflight Analysis and Conversion Engine (PACE) digital diagnostic tool.***

**Comments to the Author**

Reviewer #1

2. Has the statistical analysis been performed appropriately and rigorously?

Reviewer #1: I Don't Know

***Answer) To clarify a rationality of the statistical analysis, I added an explanation of the motivation and justification of the proposed model to the part of “Model Framework” in the revised manuscript.***

Reviewer #1: Dear authors
I have two main concerns about this manuscript:
1. The literature is neo clear as well as the critical analysis of the previous studies>
2. no comparison with the literature has been presented.
Kindly address these comments so I can review the manuscript again.
regards

***Answer) Thanks for your valuable feedback. I added a literature review to the part of “Introduction” in the revised manuscript. According to the review of previous studies, this literature review focused on the differentiated points of the proposed model from previous studies.***

 Reviewer #2

4. Is the manuscript presented in an intelligible fashion and written in standard English?

PLOS ONE does not copyedit accepted manuscripts, so the language in submitted articles must be clear, correct, and unambiguous. Any typographical or grammatical errors should be corrected at revision, so please note any specific errors here.

Reviewer #2: No

***Answer) As mentioned above, I employed a professional scientific editing service, Editage to respond the concern of Reviewer #2.***

Reviewer #2: Overall, this is a clear, concise, and well-written manuscript. The introduction is relevant and theory based. The methods are generally appropriate
As the focus was on the positive points in the work, the weaknesses should be pointed out as well and the conclusion needs an explanation of work limitations

***Answer) Thanks for your valuable feedback. I added a paragraph about the weaknesses and the limitations of this study to the part of “Discussion” in the revised manuscript.***
